# A Third Class: Functional Gibberellin Biosynthetic Operon in Beta-Proteobacteria

**DOI:** 10.3389/fmicb.2018.02916

**Published:** 2018-11-27

**Authors:** Raimund Nagel, John E. Bieber, Mark G. Schmidt-Dannert, Ryan S. Nett, Reuben J. Peters

**Affiliations:** ^1^Roy J. Carver Department of Biochemistry, Biophysics, and Molecular Biology, Iowa State University, Ames, IA, United States; ^2^Science Department, Newton Senior High School, Newton, IA, United States

**Keywords:** symbiosis, gibberellin, rhizobia, legume (nodules), evolution

## Abstract

The ability of plant-associated microbes to produce gibberellin A (GA) phytohormones was first described for the fungal rice pathogen *Gibberella fujikuroi* in the 1930s. Recently the capacity to produce GAs was shown for several bacteria, including symbiotic alpha-proteobacteria (α-rhizobia) and gamma-proteobacteria phytopathogens. All necessary enzymes for GA production are encoded by a conserved operon, which appears to have undergone horizontal transfer between and within these two phylogenetic classes of bacteria. Here the operon was shown to be present and functional in a third class, the beta-proteobacteria, where it is found in several symbionts (β-rhizobia). Conservation of function was examined by biochemical characterization of the enzymes encoded by the operon from *Paraburkholderia mimosarum* LMG 23256^T^. Despite the in-frame gene fusion between the short-chain alcohol dehydrogenase/reductase and ferredoxin, the encoded enzymes exhibited the expected activity. Intriguingly, together these can only produce GA_9_, the immediate precursor to the bioactive GA_4_, as the cytochrome P450 (CYP115) that catalyzes the final hydroxylation reaction is missing, similar to most α-rhizobia. However, phylogenetic analysis indicates that the operon from β-rhizobia is more closely related to examples from gamma-proteobacteria, which almost invariably have CYP115 and, hence, can produce bioactive GA_4_. This indicates not only that β-rhizobia acquired the operon by horizontal gene transfer from gamma-proteobacteria, rather than α-rhizobia, but also that they independently lost CYP115 in parallel to the α-rhizobia, further hinting at the possibility of detrimental effects for the production of bioactive GA_4_ by these symbionts.

## Introduction

Gibberellin A (GA) was first discovered in the eponymous fungal rice pathogen *Gibberella fujikuroi*, which eventually enabled identification of these diterpenoids in plants where they serve as hormones regulating growth and development (Hedden and Sponsel, 2015). As suggested from their production by *G. fujikuroi*, these phytohormones also affect plant-microbe interactions (De Bruyne et al., 2014). However, in bacteria GA production was first reported from nitrogen-fixing rhizobial symbionts rather than plant pathogens (Bottini et al., 2004). More recently, bacterial GA biosynthesis has been elucidated (Morrone et al., 2009; Hershey et al., 2014; Lu et al., 2015; Tatsukami and Ueda, 2016; Nagel and Peters, 2017b; Nagel et al., 2017; Nett et al., 2017b), with all the necessary genes generally found in close association within a biosynthetic operon. This operon seems to only be found in plant-associated bacteria (Levy et al., 2017). However, beyond the symbiotic alpha-proteobacteria (α-rhizobia) where the operon was originally reported and characterized (Tully and Keister, 1993; Tully et al., 1998; Keister et al., 1999; Morrone et al., 2009; Hershey et al., 2014; Tatsukami and Ueda, 2016; Nett et al., 2017b), the operon also was found to be functionally present in a diverse group of plant pathogens from the gamma-proteobacteria class (Lu et al., 2015; Nagel and Peters, 2017b; Nagel et al., 2017). Indeed, there is greater phylogenetic diversity of the operon in these phytopathogens than rhizobia (Nagel and Peters, 2017b), suggesting that bacterial GA biosynthesis originally evolved in this distinct class of bacteria.

**FIGURE 1:**
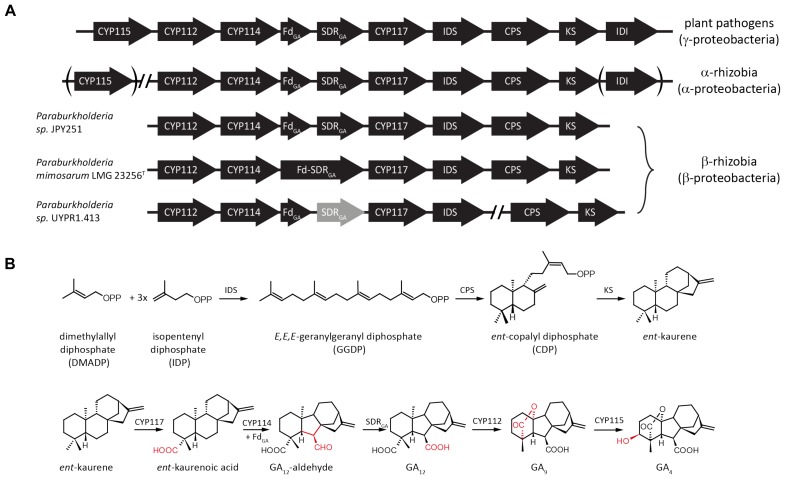
Schematic representation of bacterial gibberellin biosynthetic operon and encoded metabolic pathway. **(A)** Arrows indicate the direction of translation; abbreviations for the genes are CYP, cytochrome P450; Fd, ferredoxin; SDR, short-chain alcohol dehydrogenase/reductase; IDS, isoprenyl diphosphate synthase; CPS, copalyl diphosphate synthase; KS, ent-kaurene synthase; and IDI, isopentenyl diphosphate isomerase. **(B)** Reactions in the pathway catalyzed by the various enzymes encoded by individual genes from the operon.

The GA biosynthetic operon typically contains genes encoding eight enzymes (Figure 1). First to be characterized were the pair of diterpene cyclases that produce *ent*-kaurene from the general diterpenoid precursor (*E,E,E*)-geranylgeranyl diphosphate (GGDP). This proceeds via sequential reactions, specifically through initial production of *ent*-copalyl diphosphate (*ent*-CDP) by a CDP synthase (CPS), followed by a subsequently acting *ent*-kaurene synthase (KS) (Morrone et al., 2009; Hershey et al., 2014; Lu et al., 2015). An isoprenyl diphosphate synthase (IDS) that produces GGDP from the common isoprenoid precursors isopentenyl diphosphate (IDP) and dimethylallyl diphosphate (DMADP) is also present (Hershey et al., 2014; Nagel and Peters, 2017b). In addition, there are typically at least three cytochromes P450 (CYPs), CYP112, CYP114 and CYP117, along with a ferredoxin (Fd) and short-chain alcohol dehydrogenase/reductase (SDR). The Fd is required for the ring-contraction reaction catalyzed by CYP114, which generates GA_12_-aldehyde that is further oxidized to GA_12_ by the SDR (Nagel and Peters, 2017b; Nagel et al., 2017; Nett et al., 2017b). Beyond these common/core genes, many copies of the operon contain an isopentenyl diphosphate isomerase (IDI) that interconverts IDP and DMADP (presumably to optimize production of GGDP), and/or an additional CYP, specifically CYP115 (Nagel and Peters, 2017b; Nagel et al., 2017; Nett et al., 2017a). However, while the core operon enables biosynthesis of GA_9_, this has not been shown to have classical hormonal activity, and CYP115 is required to catalyze a subsequent hydroxylation reaction to produce bioactive GA_4_ (Figure 1).

Intriguingly, while all phytopathogens contain CYP115 in their operons, almost all α-rhizobia only have a non-functional fragment remaining, and of the less than 20% with the GA operon that also contain a full-length CYP115 gene, in every case but one it is not located in the operon (Nett et al., 2017a). Thus, most α-rhizobia can only produce the penultimate intermediate GA_9_, as has been demonstrated for *Bradyrhizobium japonicum*, although given that the operon is only expressed in nodules and the inability to otherwise distinguish between plant and bacteria produced GAs, this required isolation of bacteroids from nodules and radio-isotope feeding studies (Mendez et al., 2014). By contrast, phytopathogens can produce the bioactive GA_4_. This difference in final GA product presumably reflects their distinct relationships with plant hosts. While it has been noted that a symbiotic beta-proteobacteria (β-rhizobia) also contains the operon (Nagel and Peters, 2017b), this has not otherwise been investigated. The genome of *Paraburkholderia mimosarum* LMG 23256^T^ contains the core operon necessary for GA biosynthesis. This β-rhizobium was isolated from *Mimosa pigra* and shown to form indeterminate nodules with its host (Chen et al., 2005, 2006). Here the enzymes encoded by the operon from *P. mimosarum* LMG 23256^T^ were characterized and shown to be functionally conserved, with further phylogenetic analysis indicating not only independent acquisition of the operon from gamma-proteobacteria, but also loss of CYP115, which supports the hypothesis that direct production of bioactive GA_4_ may have a deleterious effect in the symbiotic relationship between rhizobia and their leguminous host plants.

## Materials and Methods

To find copies of the operon from newly sequenced bacterial genomes, BLAST searches were carried out using individual genes from the most divergent copy of the operon, namely that previously characterized from *Erwinia tracheiphila* (Nagel and Peters, 2017b). The results for examples found outside of the α-rhizobia are summarized in Supplementary Table S2 (along with that for the α-rhizobia used in the phylogenetic analyses reported here). Note that the bacterial genus *Burkholderia* was recently reclassified as *Paraburkholderia* (Sawana et al., 2014; Dobritsa and Samadpour, 2016).

*P. mimosarum* LMG 23256^T^ (Willems et al., 2014) was obtained from the German Collection of Microorganisms and Cell Cultures (LMG 23256^T^ = DSM21841^T^). Genes from the *P. mimosarum* operon were cloned from genomic DNA with Q5 Hot Start High-Fidelity DNA polymerase (New England Biolabs) according to the product manual with 5 μl of the high-GC-content enhancer and gene specific primers (Supplementary Table S1). The IDS, KS, CPS, and CYP112 genes were cloned into pET100/D-TOPO (Invitrogen). CYP117 was cloned into pET101/D-TOPO (Invitrogen) including a stop codon to omit the C-terminal His-Tag of the vector. CYP114 was cloned in tandem with either the Fd-SDR fusion or only the Fd into pET100. For the later construct the amino acid sequence after position 86 was changed from ET to that of the Fd from *Paraburkholderia* sp. JPY251 with the sequence ADDEAT followed by a stop codon. The CPS also was obtained as a synthetic codon optimized gene (Invitrogen) and similarly cloned into pET100. Recombinant expression, protein purification and *in vitro* enzyme assays or recombinant feeding studies, as well as organic extraction and GC-MS analysis were performed as previously described (Nagel and Peters, 2017b). Briefly, all proteins were expressed in *E. coli* BL21(Star). Cells expressing *Pm*IDS were centrifuged to collect cells, which were homogenized in MOPSO buffer pH 7.2 with 10% glycerol and 10 mM MgCl_2_. The cell lysate was incubated with 1 ml Ni-NTA Agarose (Qiagen) and *Pm*IDS was eluted with imidazole and 20 μg protein was used in 0.5 ml *in vitro* assays with 50 mM IDP and DMADP each (Sigma-Aldrich). Assays were dephosphorylated using alkaline phosphatase and extracted 3 times with equal volumes of pentane. *Pm*CPS and *Pm*KS were expressed in a 50 ml metabolic engineering setting – i.e., with an IDS from *Abies grandis* that produces GGDP and either *At*KS or *At*CPS, respectively, just as previously described for analysis of other bacterial CPSs and KSs (Morrone et al., 2009; Hershey et al., 2014; Lu et al., 2015) – for 3 days at 18°C together with pIRS, to increase flux to isoprenoids (Morrone et al., 2010). Note that neither CPS or KS alone in this setting produce *ent*-kaurene. *Pm*CYP117, *Pm*CYP114 + Fd, *Pm*CYP114 + Fd-SDR and *Pm*CYP112 were expressed alone and their respective substrates were added to the 25 ml culture and the culture was incubated at 18 °C for 3 days under shaking at 180 rpm. Cultures were extracted with 3 times with equal volumes of hexanes or ethyl acetate in case of *Pm*CYP114 + Fd, *Pm*CYP114 + Fd-SDR and *Pm*CYP112. Extracts were partially purified using silica gel columns developed with hexanes and eluted with increasing concentrations of ethyl acetate. Products of *Pm*CYP117, *Pm*CYP114 + Fd, *Pm*CYP114 + Fd-SDR and *Pm*CYP112 were methylated with diazomethane and quantified by GC-MS using a Varian GC-MS with a HP5-MS column (Agilent). The injector temperature was set at 250 °C with a helium column flow of 1.2 ml/min and 1 μl injections in split-less mode. The initial temperature of the GC oven was 50°C which was held for 3 min and increased by either 15°C/min for products of *Pm*IDS, PmCPS and PmKS or by 10°C/min for all other enzymes, until a temperature of 300 °C was reached, which was held for 3 min.

Phylogenetic analyses focused on a representative set of species and utilized nucleotide sequences spanning the core operon (i.e., from CYP112 to the KS, with CYP115 and IDI not included due to their absence in most rhizobia), including the intergenic regions. This was carried out with the nucleotide sequence of the operon, instead of the previously used concatenated protein sequences of the same region (Nagel and Peters, 2017b), as this allowed inclusion of operons with inactivating mutations (e.g., premature stop codons or frame shift mutations) as well as intergenic regions. Sequences were aligned using the Muscle algorithm in MEGA 7 (Kumar et al., 2016), and phylogenetic trees were then constructed and tested with the Maximum Likelihood Neighbor Joining and Minimum Evolution algorithms. The Tamura 3-parameter model was used with inclusion of a gamma distribution. For the Maximum Likelihood algorithm all sites were used, while for the Neighbor Joining and Minimum Evolution algorithms positions with less than 50% coverage were eliminated. The accuracy of the tree was tested via bootstrap testing with 1000 replicates each.

## Results

Beyond the core operon previously reported from *Paraburkholderia sp.* JPY251 (Nagel and Peters, 2017b), BLAST searches found two other copies in β-rhizobia. Another complete copy of the core operon was found in *P. mimosarum* LMG 23256^T^, while an incomplete copy is present in *Paraburkholderia sp*. UYPR1.413, where the operon is split between two contigs, with a transposable element found adjacent to the 3’ end of the IDS, and the SDR contains inactivating frame-shift mutations (see Figure 1A). As previously reported for *Paraburkholderia sp.* JPY251 (Nagel and Peters, 2017b), CYP115 is not found in the operon, nor elsewhere in the genome, in *P. mimosarum* or *Paraburkholderia sp.* UYPR1.413.

**FIGURE 2:**
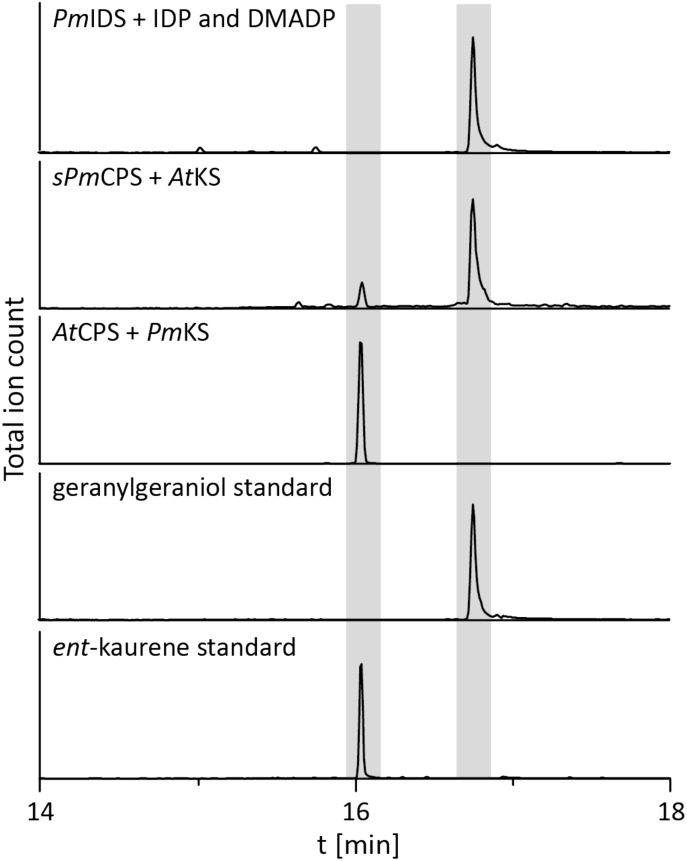
IDS, CPS, and KS enzyme activity. GC-MS chromatogram from an *in vitro* enzyme assay of *Pm*IDS with dimethyl allyl diphosphate (DMADP) and isopentenyl diphosphate (IDP) as substrates, with subsequent dephosphorylation with alkaline calf intestine phosphatase. GC-MS chromatograms of organic extracts from *E. coli* cultures expressing *Pm*KS or synthetic *sPm*CPS along with either *At*CPS or *At*KS, with additional engineering for GGDP production. GC-MS chromatograms of geranylgeraniol (dephosphorylated GGDP) and *ent*-kaurene standards are included for reference.

To determine if the operon found in the beta-proteobacteria is fully functional – i.e., enables GA production – that from *P. mimosarum* was biochemically characterized by recombinant expression in *Escherichia coli* and examination of enzymatic activity. *Pm*IDS was assessed via *in vitro* assays, which readily demonstrated the expected production of GGDP from IDP and DMADP (Figure 2 and Supplementary Figure S1). *Pm*CPS was not well-expressed in *E. coli* and no activity was observed with the purified protein *in vitro*. Activity was then assessed via a metabolic engineering approach, involving co-expression with *At*KS, the *ent*-CDP specific KS from *Arabidopsis thaliana*, along with a GGDP producing IDS from *Abies grandis* (*Ag*GGPS), as previously described (Morrone et al., 2009; Hershey et al., 2014; Lu et al., 2015). While the native gene for *Pm*CPS further did not exhibit activity in this setting either, use of a synthetic gene codon-optimized for expression in *E. coli* enabled the expected production of *ent*-kaurene (Figure 2 and Supplementary Figure S1). *Pm*KS also was assessed via a metabolic engineering approach, via co-expression with *Ag*GGPS and *At*CPS, the *ent*-CDP producing CPS from *A. thaliana*, again as previously described (Morrone et al., 2009; Hershey et al., 2014; Lu et al., 2015). These cultures similarly produced the expected *ent*-kaurene (Figure 2 and Supplementary Figure S1).

**FIGURE 3:**
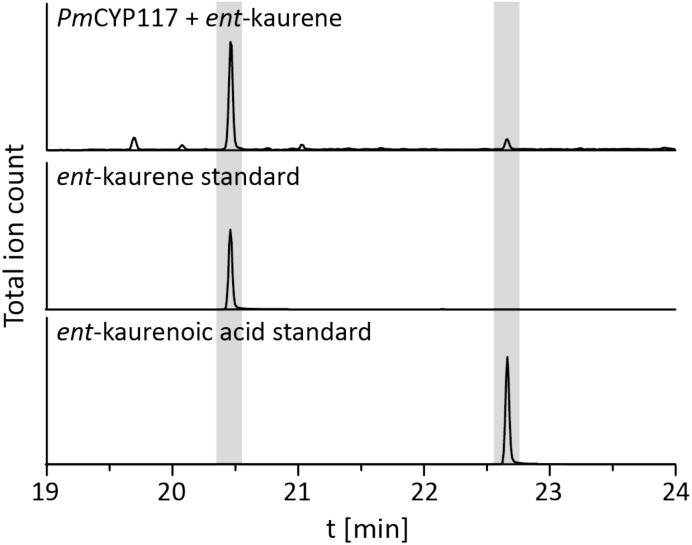
CYP117 enzyme activity. GC-MS chromatogram of the organic extract of an *E. coli* culture expressing *Pm*CYP117 and fed *ent*-kaurene. GC-MS chromatograms of *ent*-kaurene and *ent*-kaurenoic acid standards are included for reference.

**FIGURE 4:**
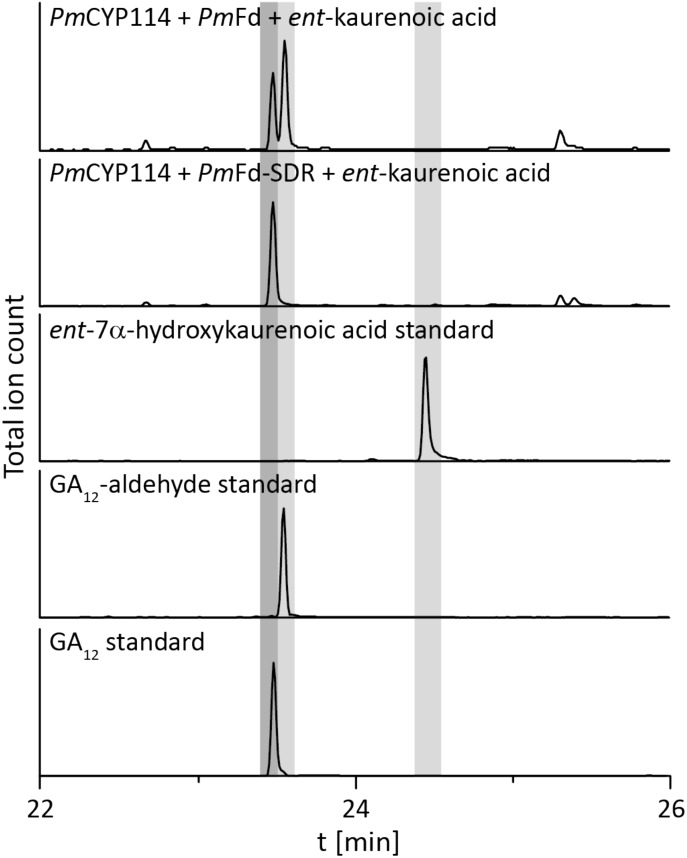
CYP114+Fd and CYP114 + Fd-SDR enzyme activity. GC-MS chromatograms from organic extracts of *E. coli* cultures expressing *Pm*CYP114 either with the artificial *Pm*Fd or the *Pm*Fd-SDR fusion protein found in *P. mimosarum* and fed *ent*-kaurenoic acid. GC-MS chromatograms of *ent*-7a-hydroxykaurenoic acid, GA_12_-aldehyde and GA_12_ standards are included for reference.

**FIGURE 5:**
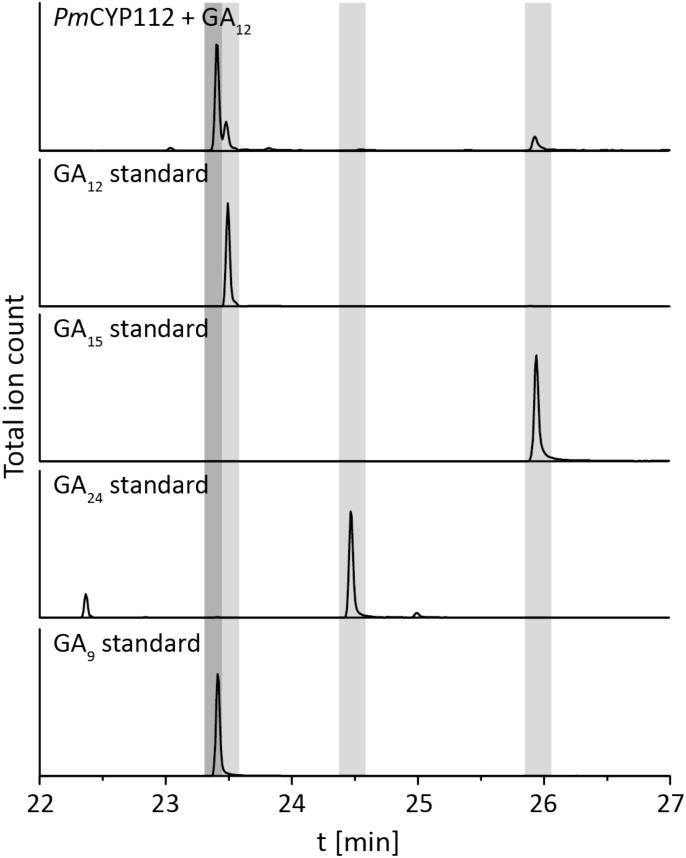
CYP112 enzyme activity. GC-MS chromatograms from organic extract of an *E. coli* culture expressing *Pm*CYP112 and fed GA_12_. Chromatograms of GA_12_, GA_15_, GA_24_, and GA_9_ standards are included for reference.

The remaining oxidative enzymes were investigated by whole-cell feeding studies. Accordingly, *ent*-kaurene was added to *E. coli* expressing *Pm*CYP117, leading to the expected conversion to *ent*-kaurenoic acid, with the intermediates *ent*-kaurenol and *ent*-kaurenal not observed (Figure 3 and Supplementary Figure S1), consistent with the previously investigated mechanism for this reaction (Nagel and Peters, 2017a). Notably, in *P. mimosarum* the genes for the Fd and SDR are fused (in-frame). Given the previously reported requirement for the Fd to enable full activity with CYP114 (Nagel and Peters, 2017b; Nagel et al., 2017; Nett et al., 2017b), two constructs were generated to evaluate the implications of this fusion for activity. First, *Pm*CYP114 was cloned into pET100 together with the fused *Pm*Fd-SDR, including the native intergenic region between CYP114 and the Fd-SDR fusion. The second construct consisted of *Pm*CYP114 and only *Pm*Fd, with introduction of a stop codon based on the sequence of the Fd in *Paraburkholderia sp.* JPY251 where the Fd and SDR are not fused. Both constructs were expressed in *E. coli* and *ent*-kaurenoic acid was added to the resulting cultures. Cultures expressing *Pm*CYP114 + *Pm*Fd (i.e., without the SDR) produced a mixture of GA_12_-aldehyde and GA_12_, while the *Pm*CYP114 + *Pm*Fd-SDR construct exclusively produced GA_12_, with the putative intermediate *ent*-7α-hydroxykaurenoic acid not observed in either case (Figure 4 and Supplementary Figure S1), consistent with previous mechanistic investigation of this reaction (Nett et al., 2016). This demonstrated the ability of the Fd to enable full CYP114 activity as a fusion protein with the SDR, as well as the expected more efficient oxidation of GA_12_-aldehyde to GA_12_ by the SDR. Finally, *E. coli* expressing *Pm*CYP112 and fed GA_12_ efficiently converted this to GA_9_, with only small amounts of the intermediate GA_15_ observed and none of the intermediate GA_24_ or the side product GA_25_ (Figure 5 and Supplementary Figure S1), again consistent with the previously investigated mechanism for this reaction (Nagel and Peters, 2018a,b).

**FIGURE 6:**
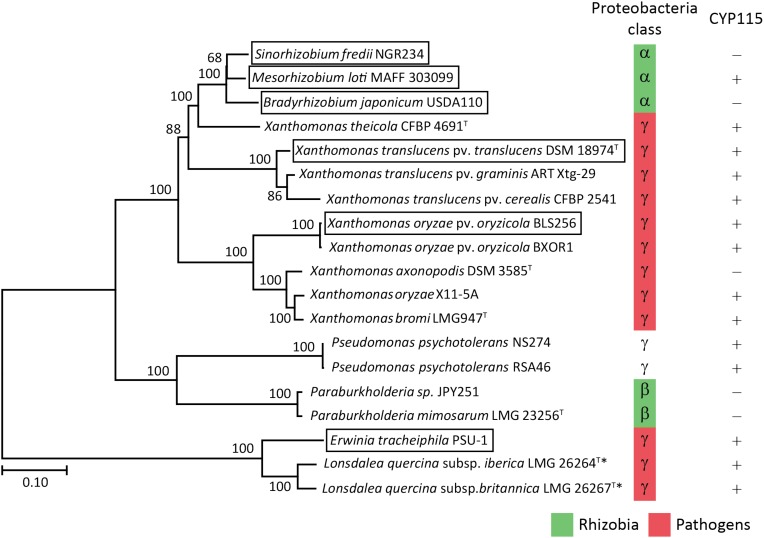
Phylogeny of the GA biosynthetic operon. Unrooted Maximum Likelihood phylogenetic tree of the operon nucleotide sequence spanning CYP112 to KS including intergenic regions. Bootstrap values were determined with 1000 replicates using MEGA 7. The scale bar represents substitutions per site and the asterisk (^∗^) indicates operons with inactivating mutations in at least one gene. The GA operon genes found within boxed species have been functionally characterized. The GeneBank accession numbers of the contig containing the operon for each of the presented bacteria can be found in Supplementary Table S2.

Despite the lack of CYP115 in the GA biosynthetic operon from β-rhizobia, which nominally resembles the operons found in α-rhizobia, it has been suggested that β-rhizobia independently obtained the operon via horizontal gene transfer from gamma-proteobacteria (Nagel and Peters, 2017b). However, this hypothesis was proposed by phylogenetic analyses limited by the single examples then available – i.e., that from *Paraburkholderia* JPY251 and the most closely related copy from a gamma-proteobacteria, *Pseudomonas psychrotolerans* (*Ps. psychrotolerans*) NS274, which includes CYP115. Given that increased numbers of species with the operon are now available, including the multiple copies noted above for *Paraburkholderia* and additional strains of *Ps. psychrotolerans*, each of which contain CYP115 (Supplementary Table S2), this phylogenetic analysis was repeated here. The results are consistent with the hypothesis that the operon in β-rhizobia is most closely related to that from *Ps. psychrotolerans* (Figure 6 and Supplementary Figure S2).

## Discussion

The biochemical results reported here demonstrate that the GA biosynthetic operon is functionally present in β-rhizobia, representing the third class of proteobacteria in which this operon can be found. However, the absence of CYP115 limits the β-rhizobia to production of the penultimate intermediate GA_9_ rather than bioactive GA_4_. While this nominally resembles previous findings in α-rhizobia, phylogenetic analysis strongly implies that the β-rhizobia independently obtained their copy of the operon from gamma-proteobacteria, as these are most closely related to the operon found in *Ps. psychrotolerans*, where CYP115 is invariably present. This further indicates that β-rhizobia also independently lost CYP115. In α-rhizobia with a full assembled genome the GA operon is invariably located within the symbiotic island or plasmid that also contains the necessary genes for nitrogen fixation (Perret et al., 1999; Gottfert et al., 2001; Sullivan et al., 2002; Gonzalez et al., 2003; Uchiumi et al., 2004; Nett et al., 2017a). However, it is unknown if the operon in beta-proteobacteria also is associated with the symbiotic island as the genome of all three species are not completely assembled and the contigs with the operon have few adjoining genes.

Beyond such implications for the origin of the operon in β-rhizobia, a number of other observations were derived from the bioinformatic analyses carried out here. For example, copies were found in *Lonsdalea quercina* subsp. *britanica* and *Lonsdalea quercina* subsp. *iberica*, which are close relatives of *Erwinia tracheiphila,* and are pathogens of oak trees (Brady et al., 2012). The *Lonsdalea* operons are also most closely related to that from *E. tracheiphila* (Figure 6 and Supplementary Figure S2), which has already been shown to be fully functional (Nagel and Peters, 2017b). However, the operons from *Lonsdalea* all are disrupted by an inactivating frame-shift mutation in CYP112, with several exhibiting additional inactivating mutations in other genes (Supplementary Table S2), suggesting that these are no longer functional, presumably reflecting a loss of selective pressure for GA production in these phytopathogens.

In the *Xanthomonas* genus, consistent with a previous report (Nagel et al., 2017), the operon is selectively present in certain pathovars of *X. oryzae*, namely *Xanthomonas oryzae* pv. *oryzicola* (all 21 sequenced genomes), but not in the other major pathovar *Xanthomonas oryzae* pv. *oryzae*, where it is not found in any of the 380 genomes currently available. By contrast, the operon is widespread in *X. translucens*, where it is present in all 48 sequenced genomes, which cover a range of pathovars, although there appears to be a premature stop codon in CYP115 in 3 of the 5 sequenced strains from *Xanthomonas translucens* pv. *poae* (Supplementary Table S2). These form a distinct cluster within the relevant clade from *X. translucens* (Langlois et al., 2017), perhaps indicating some loss of selective pressure for production of bioactive GA_4_ relative to the immediate precursor GA_9_ in this group of non-cereal phytopathogens. Regardless, it seems clear that *X. translucens* acquired the operon early, with the selective advantage provided by the production of GA_4_ leading to its widespread retention (i.e., vertical descent).

In addition, the operon was found in two new *Xanthomonas* species, *X. axonopodis* and *X. theicola*, although the operon in *X. axonopodis* appears to be missing CYP115 (Supplementary Table S2). While the division between the operons in *X. translucens* and *X. theicola* relative to *X. oryzae*, *X. bromi* and *X. axonopodis* reflects their broader phylogenic relationship (Merda et al., 2017), the numerous other species within the *Xanthomonas* genus that completely lack the operon indicate that these individually acquired the operon – i.e., via horizontal gene transfer. Consistent with this hypothesis, *X. translucens* and *X. theicola* represent a quite divergent group even within the broader *Xanthomondaceae* family (Naushad et al., 2015). Moreover, although *X. oryzae*, *X. bromi* and *X. axonopodis* all come from the same clade within this genus they do not otherwise group together (Merda et al., 2017), and the selective presence of the operon in *X. oryzae* pv. *oryzicola* versus *X. oryzae* pv. *oryzae*, as well as in *Xanthomonas* *axonopodis* DSM 3585 versus other strains of *X. axonopodis* (several of which have genome sequences available), suggests that even within this clade the operon also may be acquired by horizontal gene transfer rather than vertical descent.

Interestingly, the operon from *X. theicola* is more closely related to those from α-rhizobia than even those from *X. translucens* (Figure 6 and Supplementary Figure S2). Accordingly, while the absence of CYP115 in *X. axonopodis* nominally resembles the operon structure in α-rhizobia, this appears to represent an independent gene loss event. In particular, the presence of CYP115 in the much more closely related *X. theicola* operon supports the hypothesis that α-rhizobia originally acquired a full operon with subsequent early loss of CYP115, which contrasts with the complete gene loss observed in β-rhizobia, in that a non-functional fragment remains in almost all α-rhizobia. This further implies that direct production of bioactive GA_4_ is generally selected against in the symbiotic relationship between these rhizobia and their leguminous hosts. However, at least one α-rhizobia GA biosynthetic operon retains CYP115 (i.e., in the same position as found in gamma-proteobacteria copies), and this appears to have undergone independent gene transfer into a subset of other α-rhizobia (Nett et al., 2017a), indicating that such direct hormone biosynthesis does provide a selective advantage for at least the α-rhizobia under certain circumstances.

In conclusion, the results reported here extend our understanding of the phylogenetic range for functional acquisition of the GA biosynthetic operon beyond those previously characterized from alpha- and gamma- into beta- proteobacteria as well. Moreover, the phylogenetic analysis not only supports the hypothesis that the operon arose in the gamma-proteobacteria, but also the previously advanced hypothesis that this was acquired by independent horizontal gene transfer by both α- and β-rhizobia (Nagel and Peters, 2017b), as well as suggesting more specific origins. Strikingly, this further supports independent loss of CYP115 in both classes of rhizobia, implying that direct production of bioactive GA_4_ relative to the immediate precursor GA_9_ generally (although not universally) has deleterious effects in such symbiotic relationships. While this has been suggested to stem from suppression of the host plant defense response against microbial pathogens (Nett et al., 2017a), the actual selective pressure against retention of CYP115 remains unclear, representing an avenue for future investigation.

## Author Contributions

RN and RP designed the research and wrote the manuscript. RSN, JB, and MS-D revised the manuscript. RN, JB, MS-D, and RSN performed the research and analyzed the data.

## Conflict of Interest Statement

The authors declare that the research was conducted in the absence of any commercial or financial relationships that could be construed as a potential conflict of interest.
