# Supplementary material for: A Third Class: Functional Gibberellin Biosynthetic Operon in Beta-Proteobacteria
Source: Front Microbiol. 2018 Nov 27;9:2916. doi: 10.3389/fmicb.2018.02916 (PMC6278637; doi:10.3389/fmicb.2018.02916)
Supplement: Supplementary file 1 [file Data_Sheet_1.pdf]

Supplemental Information for:

A third class: Functional gibberellin biosynthetic operon in beta-proteobacteria

Nagel, Raimund[1]; Bieber, John E. [1;2]; Schmidt-Dannert, Mark G. [1]; Nett, Ryan S. [1]; Peters, Reuben J. [1]*

Affiliations: [1]Iowa State University, Roy J. Carver Department of Biochemistry, Biophysics, and Molecular Biology, 1210 Molecular Biology Building, Ames, IA, 50011, USA; [2]Newton Senior High School, 800 East 4th Street South, Newton, IA, 50208, USA.

Supplemental Table S1: Primer used for cloning of genes.

Primer name	Primer Sequence
IDS for	CACC ATG CAT TCC GAA CCC ACG ATG CAC
IDS rev	CTA ACG CAG ACA CGT GGC AGC C
CPS for	CACC ATG CAA TCA CTT CCC GAG CAA ATA CTC AGC
CPS rev	TTA CCG CGC CTC CCC CGG G
synthetic CPS for	CACC ATG CAG AGC CTG CCG GAA CAA ATT C
synthetic CPS rev	TTA ACG TGC TTC ACC AGG ATC ATC GG
KS for	CACC ATG CAG GCG GAA CGC GC
KS rev	TTA ACC CGT TGG CCT CCA CGA ATT CC
CYP117 for	CACC ATG AAC CTG CTG CTC AAT CCG TTC G
CYP117 rev	TCA CGA GAA GCC TAT ACG GAT GGC G
CYP114 for	CACC ATG GAG ATG CAC GAC ACT AAT TCA GAA TGT CAG G
Fd artificial stop rev	TCA AGT CGC CTC ATC GTC TGC GC
SDR rev	TTA CAC CGG CCT GGT AGC GGA TG
CYP112 for	CACC ATG CCT GAA GAA CAT CTG CCG ACG
CYP112 rev	TCA CCA GAG CAC AAG GAA TTC CTC GAA G

Supplemental Figure S1

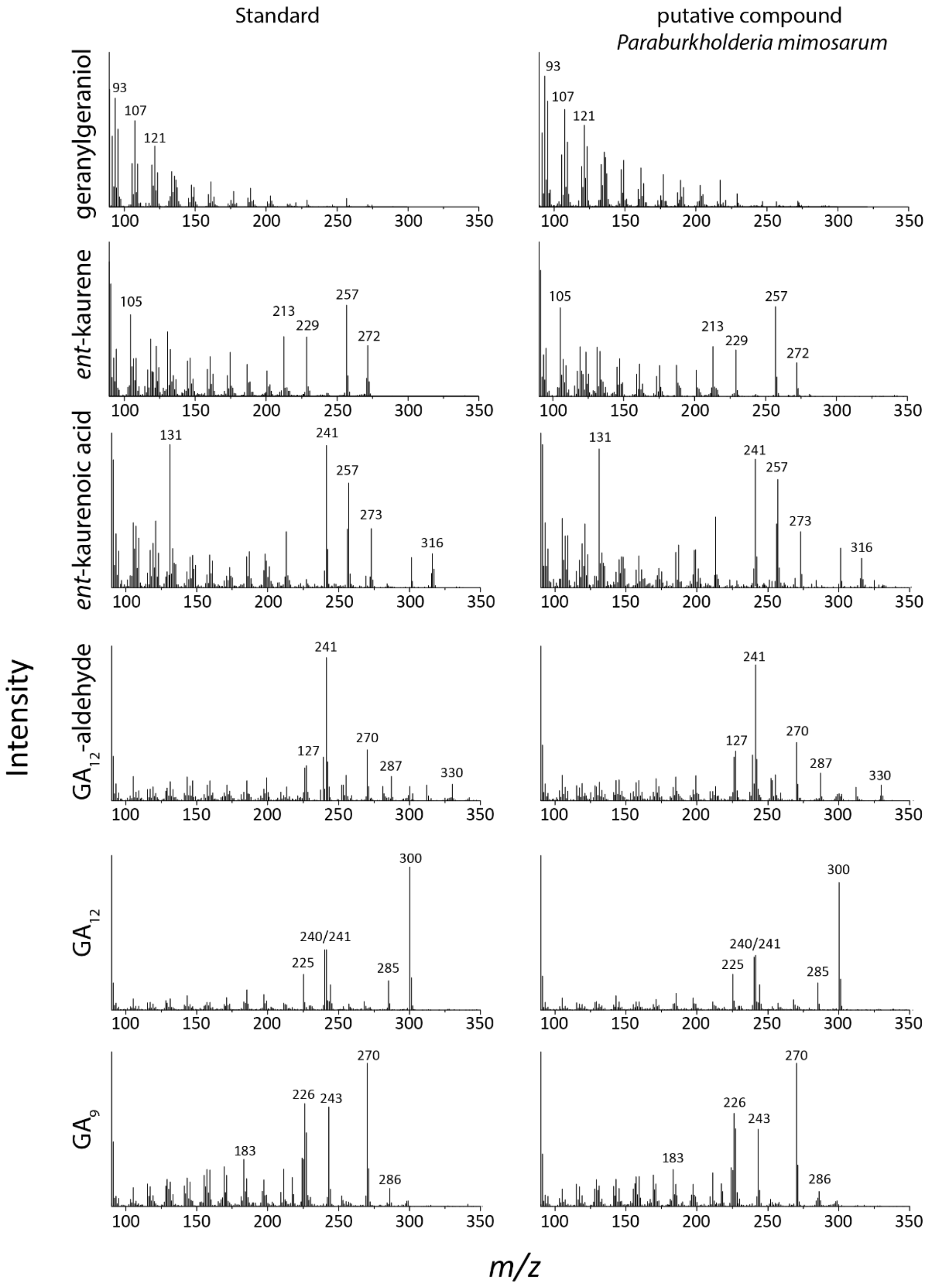

Supplemental Figure S1: Mass spectra from GC-chromatograms. Mass spectra of indicated peaks in Figure 2, 3, 4 and 5, with comparison to that of the corresponding authentic standards to confirm their identity.

Supplemental Figure S2

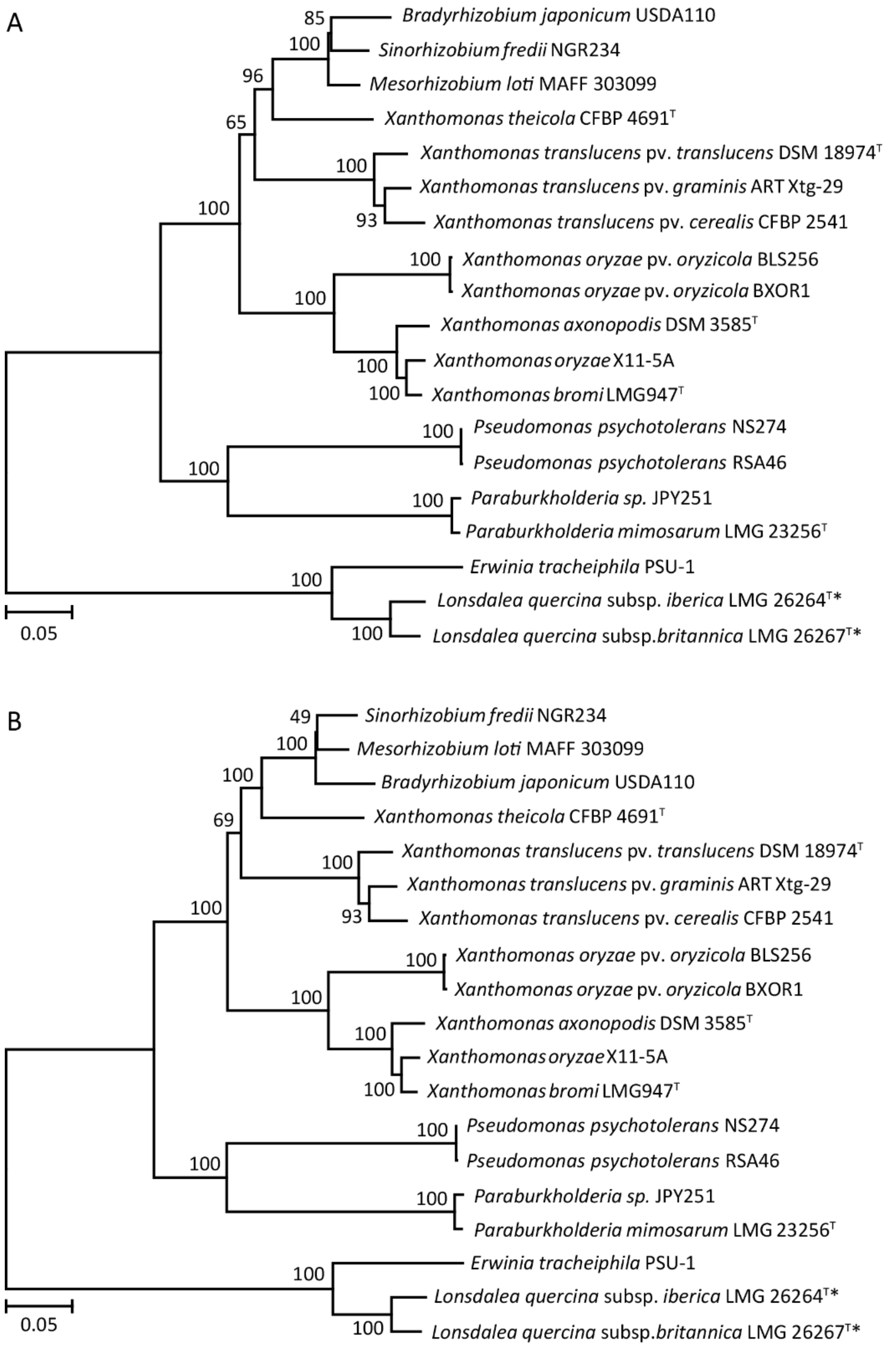

Supplemental Figure S2: Phylogeny of the GA biosynthetic operon. (A) Unrooted Neighbor Joining, (B) unrooted Minimum Evolution phylogenetic trees of the operon nucleotide sequence spanning CYP112 to KS including intergenic regions. Bootstrap values were determined with 5000 replicates using MEGA 7. The scale bar represents substitutions per site and the asterisk (*) indicates operons with inactivating mutations in at least one gene.
